# New insights of necroptosis and immune infiltration in sepsis-induced myocardial dysfunction from bioinformatics analysis through RNA-seq in mice

**DOI:** 10.3389/fcimb.2022.1068324

**Published:** 2022-12-21

**Authors:** Yan Du, Ying Zhong, Ruilin Ding, Xiaojie Wang, Fenfen Xia, Qian Zhang, Qing Peng

**Affiliations:** ^1^ Department of Cardiology, The Affiliated Hospital of Southwest Medical University, Luzhou, Sichuan, China; ^2^ Institute of Drug Clinical Trial/GCP Center, The Affiliated Hospital of Southwest Medical University, Luzhou, Sichuan, China; ^3^ Department of Endocrinology, Affiliated Hospital of Southwest Medical University, Luzhou, Sichuan, China; ^4^ Department of Infectious Diseases, Affiliated Hospital of Southwest Medical University, Luzhou, Sichuan, China

**Keywords:** sepsis, sepsis-induced myocardial dysfunction, necroptosis, immune infiltration, RNA-seq, bioinformatics analysis

## Abstract

Sepsis is a life-threatening organ dysfunction caused by dysregulated host immune response to infection. Sepsis-induced myocardial dysfunction (SIMD) is a common complication in patients with severe sepsis and is associated with increased mortality. The molecular mechanisms underlying SIMD are complex and not well characterized. Excessive inflammation due to impaired regulation of immune response is one of the major causes of SIMD. Necroptosis is a novel type of cell death that is closely related to tissue injury and inflammation. However, the role of necroptosis in SIMD is not known. Therefore, in this study, we performed an in-depth bioinformatics analysis to investigate the relationship between necroptosis and SIMD using a mouse model generated by intraperitoneal injection of lipopolysaccharide (LPS) and the underlying mechanisms. Myocardial function was assessed by echocardiography. Histopathological changes in SIMD were analyzed by hematoxylin and eosin (H&E) staining. Gene expression profiles of the heart tissues from the SIMD and control mice were analyzed by bioinformatics analysis. Transcriptome sequencing demonstrated significant differences in the expression levels of 3654 genes in the heart tissues of SIMD mice including 1810 up-regulated and 1844 down-regulated genes. The necroptosis pathway genes were significantly enriched in the heart tissues from the SIMD group mice. We identified 35 necroptosis-related differentially expressed genes (NRDEGs) including MLKL and RIPK3. Cardiomyocyte necroptosis was confirmed by qRT-PCR and western blot analysis. The expression levels of most NRDEGs showed positive correlation with the infiltration levels of mast cells, macrophages, and neutrophils, and negative correlation with the infiltration levels of B cells and plasma cells in the heart tissues of the SIMD group mice. In conclusion, this study demonstrated that necroptosis was associated with changes in the infiltration levels of several immune cell types in the heart tissues of the SIMD model mice. This suggested that necroptosis influenced SIMD development by modulating the immune microenvironment. This suggested that NRDEGs are potential diagnostic biomarkers and therapeutic targets for patients with SIMD.

## Introduction

Sepsis is a life-threatening organ dysfunction caused by dysregulated host immune response to infection (Singer et al., 2016) and is associated with high mortality rates (Prescott and Angus, 2018). Cardiac function is significantly affected during sepsis and can be lethal (Lv and Wang, 2016; Stanzani et al., 2019; Martin et al., 2019; L'Heureux et al., 2020). Epidemiological studies have shown that 10-70% of sepsis cases develop sepsis-induced myocardial dysfunction (SIMD) (Liu and Chong, 2021). The main characteristics of SIMD include dilation of the ventricle, decreased ejection fraction, and reduced contractility (Flynn et al., 2010; Martin et al., 2019). The mortality rates of patients with SIMD is approximately 2-3 times higher than for those without SIMD (Boyd et al., 2006; Joseph et al., 2017; Walley, 2018; Ehrman et al., 2018). However, the pathogenetic mechanisms underlying SIMD are not completely understood. Therefore, there is an urgent need to characterize the mechanisms underlying the pathogenesis and development of SIMD to guide clinical diagnosis and treatment that would improve clinical outcomes.

Necroptosis is a novel mechanisms of programmed cell death that is associated with several human diseases (Linkermann and Green, 2014). The cells undergoing necroptosis demonstrate characteristics of both necrosis and apoptosis. Necroptosis is induced by activating death receptors like TNF receptor 1 (TNFR1) (Yuan et al., 2019). TNFR1 activation promotes activation of the receptor-interacting protein kinase 1 (RIPK1), which subsequently phosphorylates and activates RIPK3. The RIPK1-RIPK3-mixed kinase domain-like protein (MLKL) complex is responsible for cell death *via* formation of membrane pores. Necroptosis is observed during cardiac ischemia-reperfusion injury and myocardial depression induced by oxidative stress (Soppert et al., 2018; Heckmann et al., 2019). Although necroptosis is linked to multiple human diseases (Choi et al., 2019), the potential role of necroptosis in SIMD is poorly defined.

The immune system plays an essential role in cardiac diseases including myocardial infarction, myocarditis, endocarditis, and arrhythmias (Swirski and Nahrendorf, 2018). The pathogenesis of sepsis involves severe and persistent inflammatory response after systemic activation of the immune system by the invading microbes (Delano and Ward, 2016; Venet and Monneret, 2018). Previous bioinformatics analyses have demonstrated correlation between alterations in immune infiltration and sepsis (Xu et al., 2020; Li et al., 2021). These findings suggest that immune infiltration participates in the development of SIMD.

In this study, we generated the lipopolysaccharide (LPS)-induced myocardial dysfunction model mice through intraperitoneal injections and analyzed the transcriptome changes in the cardiac tissues using bioinformatics analyses. Furthermore, we investigated the relationship between immune infiltration and necroptosis in SIMD.

## Materials and methods

### Ethics statement

All the animal experiments were performed in accordance with the National Institutes of Health Guidelines for the Care and Use of Laboratory Animals. The animal protocols were approved by the Ethics Committee of the Southwest Medical University for Animal Care and Treatment (IACUC Issue No. SWMU20220041).

### Animal grouping and modeling

Eight-to-twelve-week-old male C57BL/6J mice were maintained in a sterile facility with a 12-hour light/dark cycle, constant humidity and temperature, and free access to food and water. The animals were allowed to acclimatize for a week before conducting the experiments (Sun et al., 2018; Li et al., 2019). The mouse SIMD model was generated by an abdominal injection of 10 mg/kg LPS (a constituent of the gram-negative bacteria), which was purchased from the American Sigma Inc (Fungus: O55: B5). In the pre-trial experiments, ten mice were randomly divided into two groups. The SIMD model mice (n=5) were injected with 10 mg/kg LPS for 12 h, whereas the control mice were injected with the same volume of normal saline. After 12 h, the mice were anesthetized. Transthoracic echocardiography was performed to evaluate the cardiac function. Then, the mice were sacrificed and the heart tissues were harvested and analyzed by histopathology. The levels of IL-1β, IL-6, TNF-α, creatine kinase isoenzyme (CKMB), and cardiac Troponin-T (CTNT) were estimated in the heart tissue samples by ELISA. After confirming the success of the SIMD model, transcriptome sequencing was performed with heart tissue samples from the two groups of mice: (1) SIMD group that received intraperitoneal injections of 10 mg/kg LPS (n =3); (2) control group that received same volume of saline (n =3).

### Echocardiography analysis of cardiac function

The mice were anesthetized with 1.0-1.5% isoflurane at 12 h after LPS or saline administration. The changes in cardiac function were analyzed by echocardiography (30 MHz, VisualSonics Vevo 3100). Two-dimension (2D)-guided M-mode measurements of the internal left ventricular (LV) diameter were estimated from the short-axis view at the papillary muscles over a minimum of three heartbeats and averaged. Cardiac function parameters including ejection fraction (EF), fractional shortening (FS), left ventricular end-systolic diameter (LVEDd), and left ventricular end-diastolic diameter (LVEDs) were estimated from echocardiography.

### Collection and treatment of heart and serum samples

After measuring the cardiac function, blood samples were collected from the eyeball of the mice in a clean EP tube. The blood samples were centrifuged at 3000 rpm for 20 minutes and 4°C and the supernatant was collected as the serum. The serum samples were stored at − 80° C for further analysis. The mouse heart samples were harvested, washed with saline, and cut into two parts. One section of the mouse heart was frozen at -80°C for qRT-PCR and western blotting, whereas the other section was fixed in 4% paraformaldehyde for histopathology experiments.

### ELISA

ELISA were performed to estimate the serum levels of sepsis biomarkers, namely, IL-1β, IL-6, TNF-α, CKMB, and CTNT using the following ELISA kits from ELK Biotechnology CO., LTD, Wuhan, China according to the manufacturer’s directions: IL-1β (Cat: ELK1271), IL-6 (Cat: ELK1157), TNF-α (Cat: ELK1395), CKMB (Cat: ELK1286), and CTNT (Cat: ELK6207). The values were reported as ng/pg of total protein.

### Hematoxylin and eosin (HE) staining

Murine cardiac tissues were fixed in 10% paraformaldehyde, embedded in paraffin wax, and 4 μm thick slices were cut. After deparaffinization at 60°C, the sections were incubated twice in xylene, and dehydrated in gradient ethanol solutions. The sections were stained for 10 min with Harris hematoxylin staining solution followed by incubation with 1% HCl in ethanol for 30 sec. The slices were then washed with tap water for 15 minutes, stained with 1% eosin iron-red, and incubated with 90% ethanol. Then, the sections were washed with 95% ethanol for 1 minute, and then 3 times with xylene. Finally, the hematoxylin and eosin (H&E) stained sections were allowed to sit at room temperature for 20 min and then analyzed by light microscopy at a magnification of x400 to determine the morphological changes in the myocardium.

### RNA sequencing

Total RNA from the myocardial tissues was extracted using the TRIzol reagent and DNase I (Takara) according to the manufacturer’s instructions. The samples were assessed by 1% agarose gel to estimate RNA degradation and contamination. RNA quality was determined using a 2100 Bioanalyzer (Agilent Technologies, Santa Clara, CA, USA) and quantified using the NanoDrop ND-2000 spectophotometer (Nanodrop Technologies Inc., Wilmington, USA). High-quality RNA samples (OD260/280 = 1.8~2.2; OD260/230 ≥ 2.0; RIN ≥ 8.0; 28S: 18S ≥ 1.0) were used for sequencing. RNA samples were purified, reverse transcribed, and cDNA libraries were constructed and sequenced by the Shanghai Majorbio Bio-pharm Biotechnology Co., Ltd. (Shanghai, China). The RNA sequencing datasets from the current study are available in online repositories (https://www.ncbi.nlm.nih.gov/, PRJNA889757).

### Identification of necroptosis-related differentially expressed genes (NRDEGs)

R language package “limma (v 3.48.3)” was used to screen differentially expressed genes (DEGs) with adjusted p ≤ 0.05 and |log2FC| ≥ 1 as threshold parameters (Ritchie et al., 2015). We identified 159 necroptosis-related genes in the Kyoto Encyclopedia of Genes and Genomes (KEGG) Pathway database (https://www.genome.jp/dbget-bin/www_bget?pathway+hsa04217). NRDEGs were identified using the online Venn diagram tool(https://bioinfogp.cnb.csic.es/tools/venny/index.html).

### Gene set enrichment analysis (GSEA)

The DEGs were analyzed by GSEA to identify the underlying biological functions using the c2 (c2.cp.kegg.v7.5.1.entres.gmt) gene set from the “clusterProfile” R package (Subramanian et al., 2005). p <0.05 was considered as statistically significant.

### Construction of protein-protein interaction (PPI) network and functional enrichment analysis of NRDEGs

PPI network of the NRDEGs was visualized using the STRING database (https://string-db.org/) and the Cytoscape software (version 3.9.0) (Cline et al., 2007; Szklarczyk et al., 2015). The CytoNCA plugin of the Cytoscape software (Tang et al., 2015) was used to identify the hub genes of the PPI network. Gene Ontology (GO) and Kyoto Encyclopedia of Genes and Genomes (KEGG) pathway enrichment analyses were performed using the “clusterProfiler” R package. The GO enrichment categories related to biological process (BP), cellular component (CC), and molecular function (MF), and the KEGG pathways were visualized as bubble charts using the “enrichplot” package in R.

### Immunohistochemistry

The paraffin-embedded murine heart tissues were analyzed by immunohistochemistry. The cryostat cut murine heart sections (3 μm) were washed 3 times with PBS, incubated with 3% H_2_O_2_ solution for 30 min, and blocked with 5% BSA for 30 min. Then, the heart tissue sections were incubated overnight at 4°C with the following primary antibodies: rabbit anti-human RIPK3(1:150, novus, NBP1-77299), Caspase-1(1:200, novus, 22915-1-AP), GSDMD (1:150, Affinity, AF4012), and mouse anti-human MLKL (1:200, novus, 66675-1-Ig). The sections were then incubated for 30 minutes at room temperature with the following HRP-conjugated secondary antibodies: rabbit anti-goat (1:200, Servicebio, GB23204), goat anti-rabbit (1:200, Aspen, AS-1107), goat anti-mouse (1:200, Aspen, AS-1106), and goat anti-rat (1:200, Servicebio, GB23302). The samples were incubated with DAB and hematoxylin II solutions for 10 minutes each, and then incubated with the Bluing reagent for 4 minutes. The sections were then dehydrated and mounted on slides using the mounting medium. Images were captured using an Olympus Ix51 microscope (Olympus America, Melville, NY, USA) and analyzed using the CaseViewer software (version 2.3).

### Quantitative real-time PCR

Total RNA was extracted from the heart tissues using the TRIzol reagent (ELK Biotechnology) following the manufacturer’s instructions. Then, RNA samples (2µg) were reverse transcribed into cDNA using the EntiLink 1st Strand cDNA Synthesis Super Mix (ELK Biotechnology, EQ031). Then, qRT-PCR was performed in the QuantStudio 6 Flex System (Life Technologies) with the cDNA samples using the EnTurbo SYBR Green PCR SuperMix (ELK Biotechnology, EQ001). Glyceraldehyde 3-phosphate dehydrogenase (GAPDH) was used as the internal control. The qPCR primers (**Table 1**) were designed and synthesized by HY cell biotechnology (Wuhan, China). The relative gene expression levels were analyzed using the 2^−ΔΔCt^ method.

**Table 1:** PCR primers for quantitative real-time PCR.

Genes	Forward (5’-3’)	Reverse (5’-3’)
RIPK3	AACTGAAGGAGTTAATGATTCATTG	TGCTGAGACAGATAATGCTTTACCT
MLKL	ATCAGCCGGACAGCAAAGAG	GAATCACAGCCTTCAAATGGG
Caspase8	TATCCTATCCCACGGTGACAAG	GTTACTTCCTTGGCAAGCCTG
IL-1β	GGACCCATATGAGCTGAAAGC	CATCTTCTTCTTTGGGTATTGCT
TLR3	ATCAAATCCACTTAAAGAGTTCTCC	TGTTGTACTCCAGAGACAGATACCT
TNFα	TCCCCAAAGGGATGAGAAGTT	GAGGAGGTTGACTTTCTCCTGG
Stat1	TGGGCGTCTATCCTGTGGTAC	CAGCATGCTCAGCTGGTCTG
Tradd	CAAGAAGAAGGTGGCAATATACAAG	TTCTGGGCTAAGATGTAATTCAAAC
Fas	ATCTGGGCTGTCCTGCCTCT	TTATCAGTTTCACGAACCCGC
Nlrp3	TCTCAAGTCTAAGCACCAACCG	CGAAGCAGCATTGATGGGAC
GAPDH	TGAAGGGTGGAGCCAAAAG	AGTCTTCTGGGTGGCAGTGAT

### Western blotting

The frozen heart tissue samples from 6 mice (3 LPS mice and 3 control mice) were cut into small pieces and rinsed with pre-chilled phosphate-buffered saline to remove red blood cells and other interfering substances. The heart tissues were incubated with protein lysis buffer and protease inhibitors in cold. The extracted samples were centrifuged at 12,000 ×g for 5 min at 4°C, and the supernatants were collected as total protein extracts. The protein concentrations of the samples were estimated using the BCA Protein Assay Kit (AS1086, ASPEN, Wuhan, China). Equal amounts of total protein samples in 5X Protein loading buffer (AS1011, ASPEN, Wuhan, China) were separated on an SDS-PAGE. The separated proteins were transferred onto polyvinylidene difluoride (PVDF) membranes (Millipore, IPVH00010) using a wet blotting apparatus (Beijing Liuyi Biotechnology Co., Ltd). The membranes were blocked with 5% skimmed milk for 1 h. Then, the membranes were incubated overnight at 4°C with the following primary antibodies: anti-MLKL antibody (1:2,000, Abcam, ab243142), anti-RIPK3 (1:1000, Proteintech, 17563-1-AP), anti-TNFα antibody (1:1,000, Proteintech, 17590-1-AP), anti-Caspase8 (1:2,000, Proteintech, Abcam, ab108333), anti-IL-1β antibody (1:500, affbiotech, AF5103), and anti-TLR3 (1:500, Abcam, ab62566). After washing, the blots were incubated with the following HRP-conjugated secondary antibodies: Goat anti Rabbit (1:10000, ASPEN, AS1107); Goat anti-Mouse (1:10000, ASPEN, AS1106); Rabbit anti-Goat (1:10000, ASPEN, AS1108); Goat anti Rat (1:10000, ASPEN, AS1093); Rabbit anti Sheep (1:10000, ASPEN, AS1245). The blots were washed with 1X TBST and incubated with the ECL chemiluminescent substrate. The blots were processed using a luminescent imaging workstation. The density of the protein bands were measured using the AlphaEaseFC software (Alpha Innotech). The Canon LiDE110 scanner was used to measure the total protein levels and used as the loading control.

### Analysis of the immune microenvironment

Multiple algorithms from the IOBR R package (Zeng et al., 2021) were used to determine the immune microenvironment in the heart tissues of the control and SIMD group mice. The CIBERSORT deconvolution algorithm estimates the cellular composition of complex tissues based on gene expression profiles by comparing the expression levels of gene sets from different subsets of immune cells in the tissue samples (Newman et al., 2015; Chen et al., 2018a). CIBERSORT can be used to estimate the abundance of 22 different types of immune cells using 1000 permutations. xCell employs machine learning algorithm based on gene signatures from thousands of different cell types that map the cellular heterogeneity of tissue expression profiles. TIMER uses an inverse convolution approach for estimating the proportion of six different immune cell types in tissue samples. Next, we obtained a matrix of the immune cell types and visualized the results using the “ggplot2” R package. The differences in the immune cell infiltration between control and SIMD groups were observed using the “ggpubr” R package.

### Statistical analysis

R software version 4.2.0 was used to perform statistical analysis. Quantitative data was expressed as mean ± standard deviation and compared using the unpaired t-test. GraphPad Prism version 7.0 (GraphPad Prism software, San Diego, CA, USA) was used for statistical data analysis. P <0.05 was considered statistically significant.

## Results

### LPS-induced mice demonstrate significant inflammation and cardiac damage

**Figure 1:**
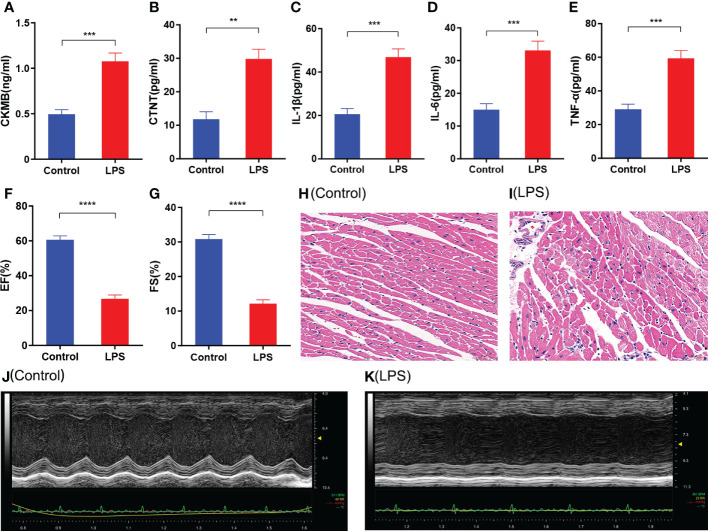
Characterization of cardiac injury and inflammatory response in the sepsis-induced myocardial dysfunction model mice generated by LPS treatment. **(A-E)** ELISA assay results show the levels of **(A)** CKMB, **(B)** CTNT, **(C)** IL-1β, **(D)** IL-6, and **(E)** TNF-α in the heart tissues of the SIMD and control groups of mice. **(F, G)** Echocardiographic data results show the **(F)** left ventricle ejection fraction (LVEF) and **(G)** left ventricle fractional shortening values for the SIMD and control groups of mice. **(H, I)** Representative images show H&E stained sections of heart tissues from the SIMD and the control groups of mice. Scale bar: 50μm. **(J, K)** Representative echocardiographic images of the SIMD and the control groups of mice. The columns represent the mean values, and the error bars represent the standard error of the mean (n = 5). *p < 0.05, **p < 0.01, ***p < 0.001, ****p < 0.0001.

The LPS group mice showed higher levels of CKMB and CTNT compared with the control group (**Figures 1A, B**). Furthermore, LPS group mice showed significantly higher levels of pro-inflammatory cytokines such as IL-1β, IL-6, and TNF-α compared to the control group (**Figures 1C-E**). Echocardiography data showed lower EF (%) and FS (%) in the LPS group mice compared with the control group mice (**Figures 1F, G**). Furthermore, histopathology data showed significant inflammatory cell infiltration in the heart tissues of mice from the LPS group compared to those from the control group (**Figures 1H, I**). The echocardiography data (**Figures 1J, K**) was consistent with the histopathological changes. These results demonstrated that inflammatory cell infiltration in the heart tissues of the LPS group mice correlated with reduced myocardial contractility.

### Identification of DEGs and NRDEGs

**Figure 2:**
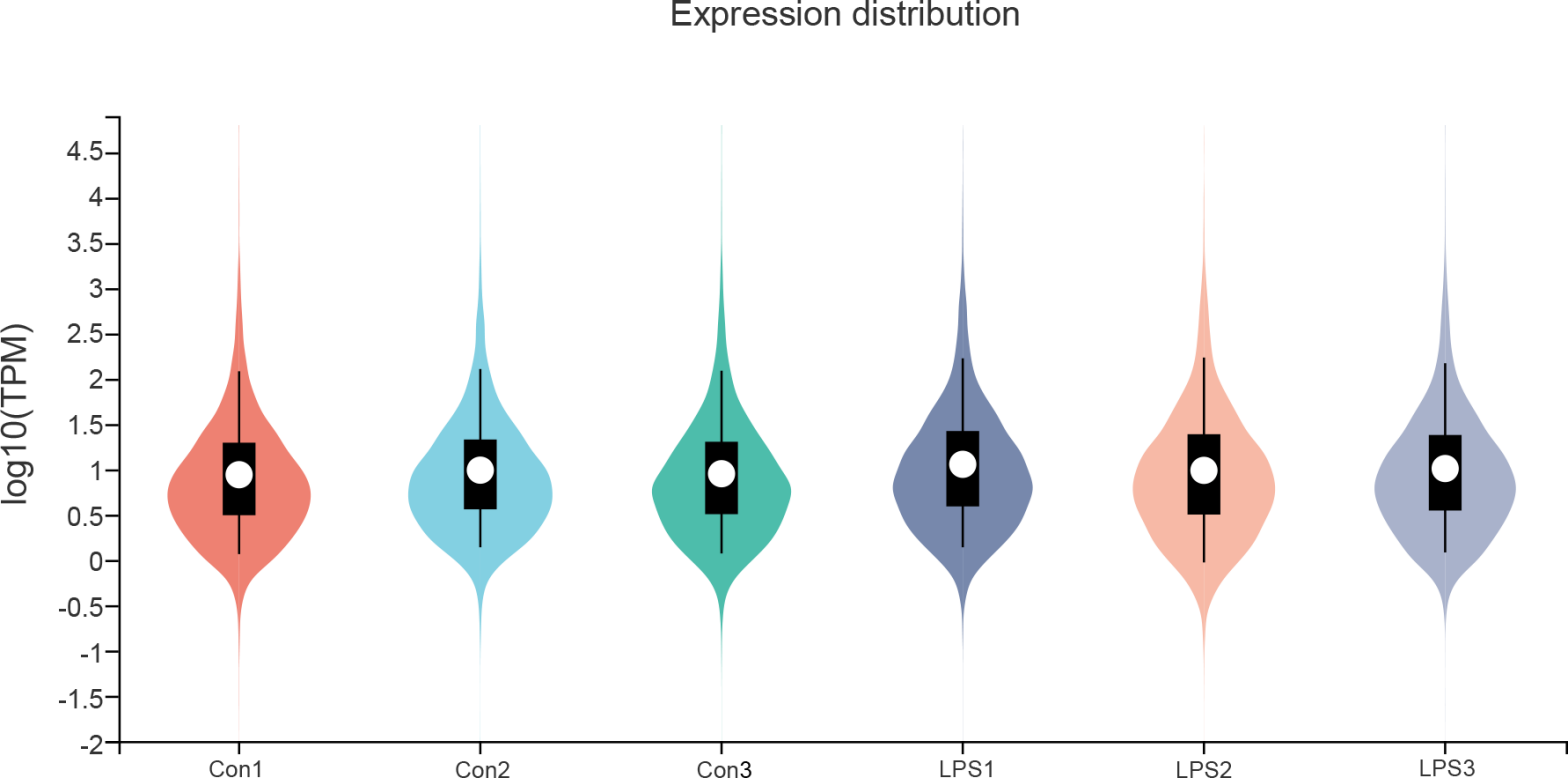
RNA sequencing data normalization for bioinformatics analysis. The violin plot shows the TPM normalization of RNA sequencing data for the control and SIMD groups.

**Figure 3:**
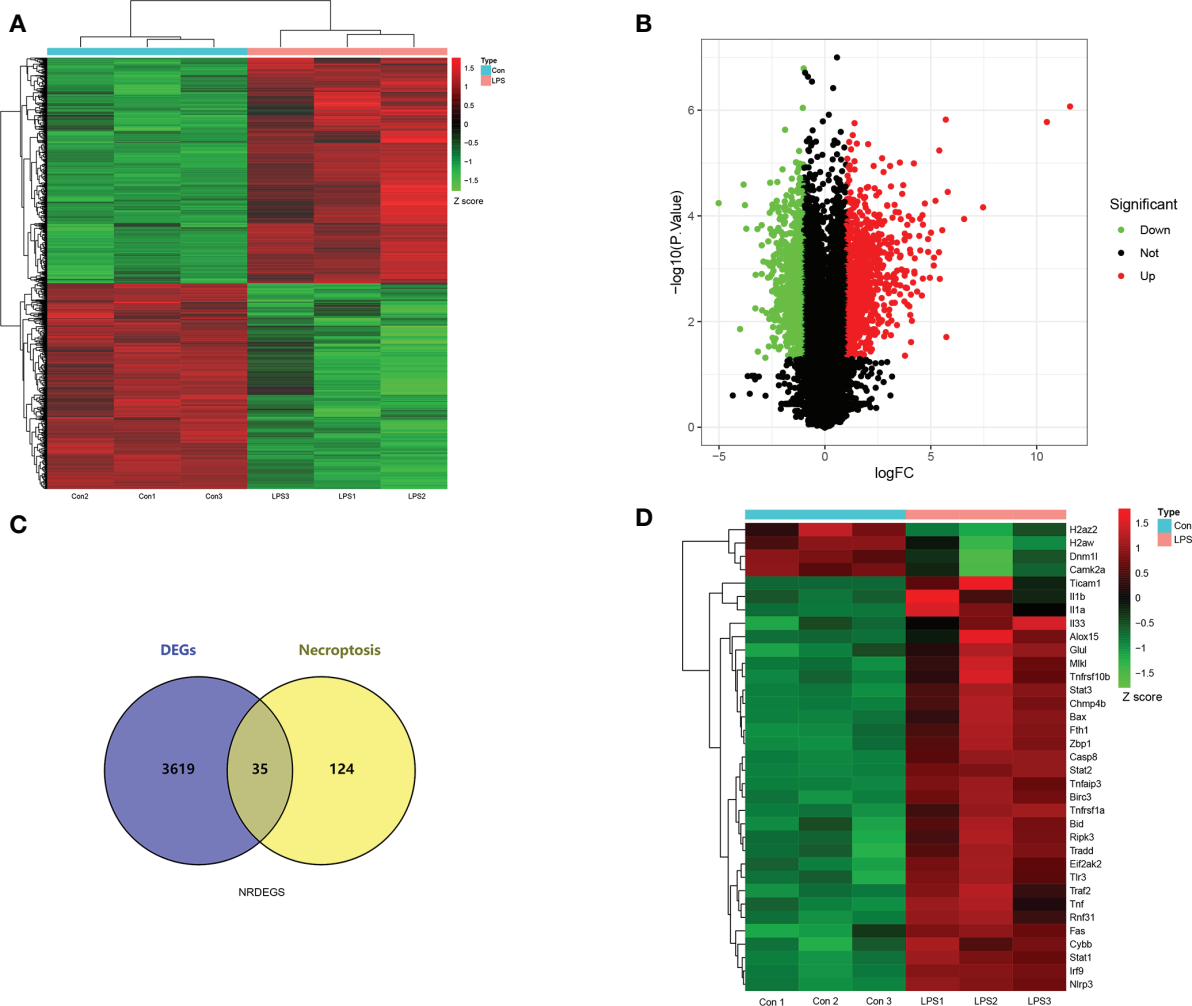
Screening of necroptosis-related differentially expressed genes (NRDEGs) in SIMD. **(A)** Heatmap shows the relative expression of the DEGs in the control and SIMD groups. Red denotes up-regulated genes, and green denotes downregulated genes. **(B)** Volcano map shows the RNA sequencing results. The green dots represent downregulated genes; the red dots represent up-regulated genes; the black dots represent unchanged genes. **(C)** Venn diagram shows the overlap between DEGs and necroptosis-related genes in the Kyoto Encyclopedia of Genes and Genomes pathway database. **(D)** Clustered heatmap of the NRDEGs.

The gene expression data of the control and SIMD mouse heart tissues was log_2_ transformed and annotated (**Figure 2**). The two data sets were uniform and comparable. The high-quality RNA-sequencing data was used for functional analysis. We first analyzed differentially expressed genes (DEGs) between the control and LPS groups and identified 3654 DEGs including 1810 up-regulated and 1844 down-regulated genes (**Figures 3A, B**). For further analyses, we identified 35 NRDEGs, which are the intersection of DEGs on 159 necroptosis-related genes including 31 up-regulated and 4 down-regulated NRDEGs as shown in the Venn diagram (**Figure 3C**). The expression heatmap of the NRDEGs is shown in **Figure 3D**.

### Gene set enrichment analysis of the DEGs

**Figure 4:**
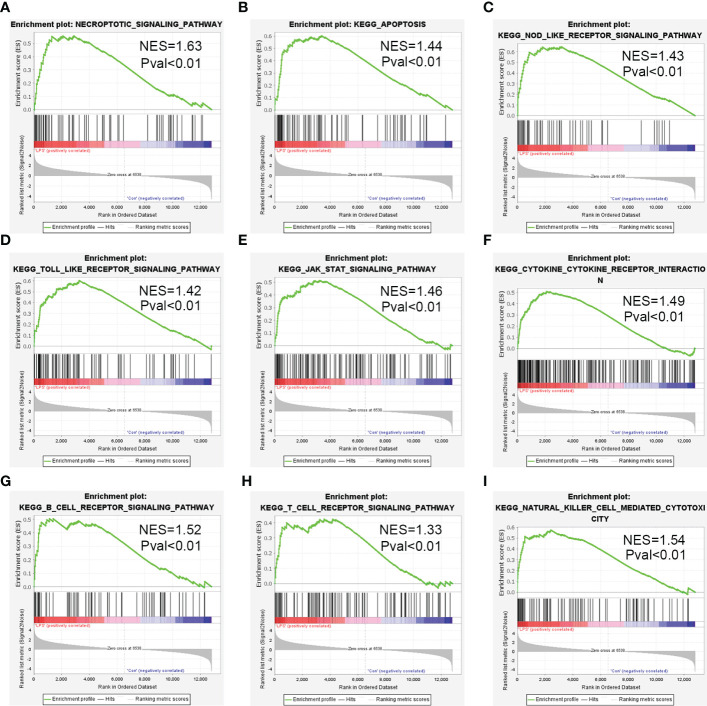
Enrichment plots from the gene set enrichment analysis (GSEA) for the highly enriched pathways in SIMD. **(A)** Necroptosis signaling pathway. **(B)** Apoptosis signaling pathway. **(C)** NOD-like receptor signaling pathway. **(D)** Toll-like receptor signaling pathway. **(E)** JAK-STAT signaling pathway. **(F)** Cytokine-Cytokine receptor interaction. **(G)** B-cell receptor signaling pathway. **(H)** T-cell receptor signaling pathway. **(I)** Natural killer cell mediated cytotoxicity.

The DEGs were evaluated by GSEA and 173 enriched KEGG signaling pathways were identified. As shown in **Figure 4A**, necroptosis pathway was significantly enriched and up-regulated (normalized enrichment score = 1.63, p-value <0.01) in the heart tissues of the SIMD group mice. GSEA data showed significant enrichment of pathways such as apoptosis, NOD-like receptor signaling pathway, Toll-like receptor signaling pathway, JAK-STAT signaling pathway, cytokine-cytokine receptor interaction, and B-cell receptor signaling pathway, T-cell receptor signaling pathway and natural killer cell mediated cytotoxicity in SIMD (**Figures 4B-I**).

### Protein-protein interaction network analysis of the NRDEGs

**Figure 5:**
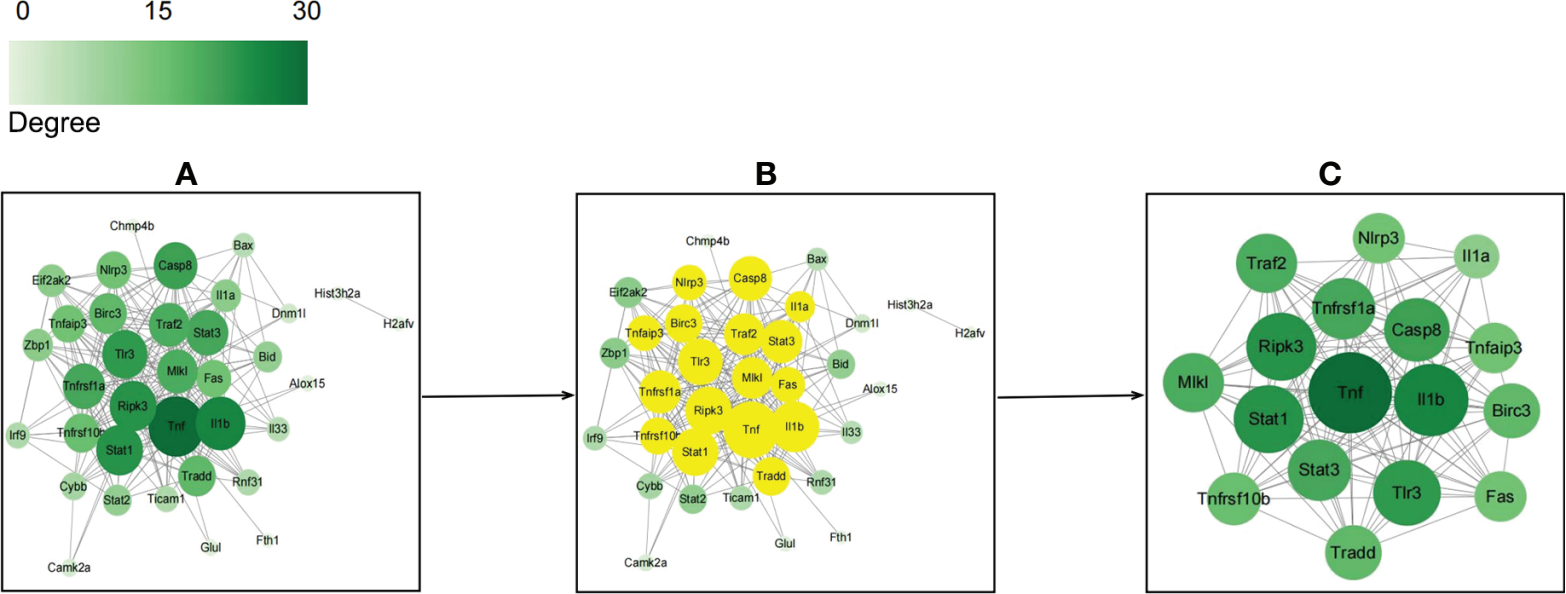
Protein-protein interaction networks of necroptosis-related differentially expressed genes (NRDEGs) based on STRING and Cytoscape database analyses. **(A)** PPI network of NRDEGs shows 218 edges and 35 nodes. **(B)** The top 17 hub NRDEGs based on CytoNCA. **(C)** The center of the NRDEGs shows 121 edges and 17 nodes.

The PPI network of NRDEGs was constructed using the STRING online tool and consisted of 35 core proteins and 218 edges (**Figure 5A**). Furthermore, CytoNCA plug-in of the Cytoscape software identified 17 hub genes with high degree among the NRDEGs (**Figure 5B**). The 17 hub NRDEGs were *TNF, TNFRSF1α, IL-1β, RIPK3, CASPASE8, STAT1, STAT3, TLR3, MLKL, TRADD, NLRP3, FAS, TRAF2, BIRC3, TNFRSF10β, TNFAIP3*, and *IL1a*. In the PPI network, we identified a cluster with 17 nodes and 121 edges, with *TNF, TNFRSF1α, IL-1β, RIPK3, CASPASE8, STAT1, STAT3*, and *TLR3* occupying the core center of the module (**Figure 5C**).

### Gene ontology and KEGG pathway analyses of the NRDEGs

**Figure 6:**
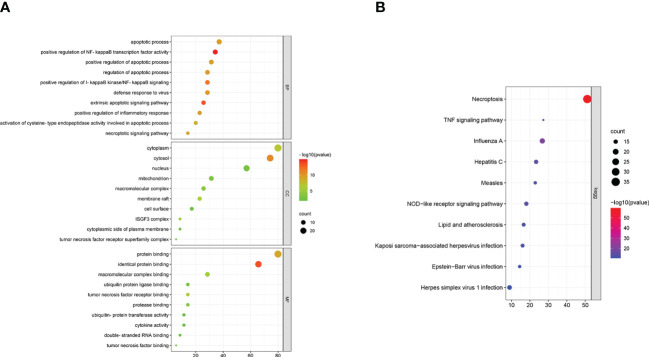
Enriched GO terms and KEGG pathways based on functional enrichment analyses of the necroptosis-related differentially expressed genes (NRDEGs). **(A)** Enriched BP, MF, and CC terms in the GO enrichment analysis of NRDEGs. **(B)** Enriched KEGG pathways represented by the NRDEGs.

Gene Ontology (GO) and KEGG pathway enrichment analysis was performed using the DAVID online database to determine the biological functions and pathways related with the NRDEGs. The enriched GO terms were classified into BP, CC, and MF ontologies. NRDEGs were enriched in BP terms such as necroptotic signaling pathway, apoptotic process, positive regulation of I-kappaB kinase/NF-kappaB signaling, inflammatory response, and cytokine-mediated signaling pathway (**Figure 6A**). NRDEGs were enriched in CC terms such as cytoplasm, cytosol, membrane raft, mitochondrion, nucleus, cell surface, and ISGF3 complex (**Figure 6A**). NRDEGs were enriched in MF terms such as protein binding, tumor necrosis factor binding, and cytokine activity (**Figure 6A**). NRDEGs were also enriched in KEGG pathways such as necroptosis, apoptosis, TNF signaling pathway, NOD-like receptor signaling pathway, innate immune response, cytokine stimulus-response, inflammatory response, and lipid and atherosclerosis (**Figure 6B**).

### Necroptosis is activated in sepsis-induced myocardial dysfunction

**Figure 7:**
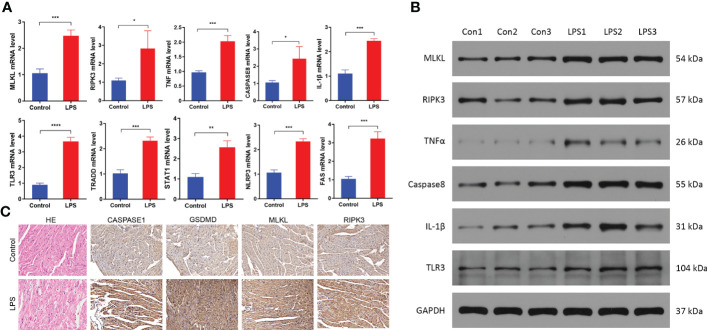
Necroptosis is activated in the heart tissues of the sepsis-induced myocardial dysfunction model mice. **(A)** QRT-PCR analysis results show the relative expression levels of TNF, IL-1β, MLKL, RIPK3, CASPASE8, TLR3, TRADD, STAT1, NLRP3 and FAS mRNAs in the heart tissues from the control and SIMD group mice (n = 3 per group). GAPDH was used as the internal control and used for normalizing the mRNA expression data. **(B)** Western blot analysis results show the expression levels of TNFα, IL-1β, MLKL, RIPK3, CASPASE8, and TLR3 proteins in the heart tissues from the control and SIMD group mice (n = 3 per group). GAPDH was used as the loading control. **(C)** Representative images show the immunohistochemical analysis of CASPASE1, GSDMD, MLKL, and RIPK3 protein levels in the heart tissues from the control and SIMD group mice. Scale bar: 50μm. Data are presented as mean ± SEM. *p < 0.05, **p < 0.01, ***p < 0.001, ****p < 0.0001.

Next, we validated the bioinformatics analysis by performing qRT-PCR and western blotting analysis of heart tissue samples from the SIMD and control group mice. QRT-PCR analysis showed significantly higher mRNA expression levels of TNFα, IL-1β, MLKL, RIPK3, CASPASE8, TLR3, TRADD, STAT1, NLRP3, and FAS in the heart samples of the SIMD group mice compared to those from the control group mice (**Figure 7A**). Western blot analysis demonstrated significantly higher expression levels of TNFα, IL-1β, MLKL, RIPK3, CASPASE8, and TLR3 proteins in the heart samples from the SIMD group mice compared to the control group mice (**Figure 7B**). The results showed higher expression of the NLRP3 inflammasome-related genes, such as IL-1β And NLRP3 in the heart tissues samples from the SIMD group mice. Previous studies have reported that RIPK3-MLKL signaling promotes inflammation by activating the NLRP3 inflammasome (Chen et al., 2018b; Guo et al., 2019). In the SIMD group, immunohistochemistry staining showed higher expression of RIPK3, MLKL, Caspase1, and Gsdmd (markers of activated NLRP3 inflammasome) in the myocardial tissue and necroptosis in the myocardial cells (**Figure 7C**). These data suggested that necroptosis and NLRP3 inflammasomes play a significant role in the development of SIMD.

### Necroptosis-related genes are associated with immune cell infiltration in SIMD

**Figure 8:**
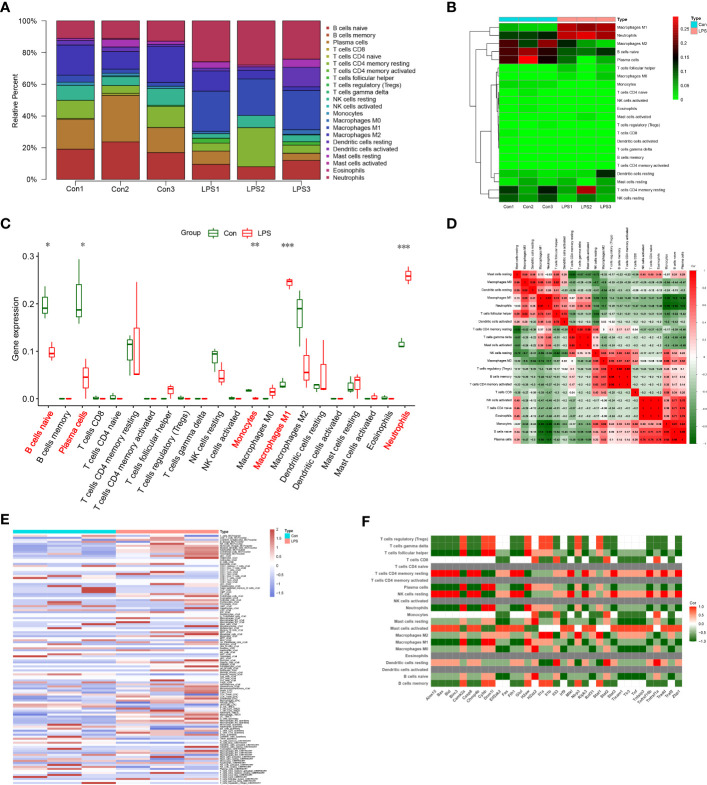
Immunocyte infiltration changes in the heart tissues of the SIMD group mice. **(A)** CIBERSORT analysis results show the proportions of 22 different immune cell types in the heart tissues of the control and SIMD group mice. **(B)** Heatmap of the 22 different immune cell types in the heart tissues of the control and SIMD group mice. **(C)** Differences in the infiltration levels of different immune cell types between the control and SIMD group mice. Red boxes denote SIMD samples, green boxes denote SIMD samples. **(D)** Cor-heatmap shows the relationship between the 22 different immune cell types. **(E)** The thermogram shows the variation in the abundance of different immune cell types in the control and SIMD groups based on multiple algorithms. **(F)** The correlation between differentially expressed necroptosis-related genes and different types of immune cells in SIMD. *p < 0.05, **p < 0.01, ***p < 0.001.

Functional enrichment analyses demonstrated that several immune-related pathways were highly expressed in the SIMD group. Therefore, we used the CIBERSORT algorithm to analyze differences in the infiltration levels of immune cells between the SIMD and control heart tissue samples. The bar plot and heat map in **Figures 8A, B** show the infiltration levels of 22 immune cell types in the heart tissue samples of the SIMD and control group of mice. The heart tissues of the SIMD group mice showed higher infiltration levels of monocytes, M1 macrophages, and neutrophils, lower infiltration levels of naive B cells and plasma cells (**Figure 8C**). The cor-heatmap in **Figure 8D** shows correlations between the 22 main types of immunocytes in SIMD. The differences in the immune cell infiltration ratios between the two groups (SIMD and control) were also analyzed using the xCell and TIMER algorithms and the results are shown in **Figure 8E**. Overall, the data showed significant differences in the infiltration levels of different immune cell types between the two groups. The results from different algorithms showed consistent changes in the immune microenvironment of the heart tissues in the SIMD group mice.

Furthermore, we estimated the correlation between NRDEGs and immune cell infiltration levels. The expression levels of most NRDEGs showed positive correlation with the infiltration levels of mast cells, macrophages, and neutrophils, and negative correlation with the infiltration levels of B cells, CD8^+^ T cells, monocytes, eosinophils, DC cells, NK cells, and most T cell subtypes (**Figure 8F**). These results demonstrated that immune cell infiltration was clinically significant for the development of SIMD. These data demonstrated that necroptosis regulated the immune microenvironment in SIMD.

## Discussion

SIMD is caused by excessive inflammatory response to infection because of impaired regulation of the immune system. The pathogenesis of SIMD is complex and challenging to treat (Zhang et al., 2020). In our study, LPS-induced myocardial dysfunction caused significant increase in the levels of several pro-inflammatory cytokines such as TNF-α and IL-1β. Activation of neutrophils plays an integral role in myocardial dysfunction and damage (Ferrante, 1992). The infiltration of immune cells is closely linked to sepsis, and the inflammatory cytokines are the main mediators of sepsis (Xu et al., 2020). LPS activates multiple programmed cell death mechanisms in the cardiomyocytes including pyroptosis, autophagy, and apoptosis (Kayagaki et al., 2015; Cao et al., 2021; He et al., 2021). However, the relationship between necroptosis and myocardial dysfunction during sepsis is not well understood. Our results demonstrate the relationship between inflammation, necroptosis, and immune response in SIMD.

The mechanism of necroptosis in SIMD is complex and unclear. Therefore, to investigate the role of necroptosis-related genes in the pathogenetic mechanisms related to SIMD, we generated the LPS-induced myocardial dysfunction model in mice. Bioinformatics analyses of the RNA sequencing data demonstrated activation of necroptosis, with differential expression of more than 35 necroptosis-related genes including RIPK3 and MLKL. The bioinformatics results were confirmed by immunohistochemistry, qRT-PCR and western blot analyses. Furthermore, the relationship between NRDEGs and immunocyte infiltration in SIMD was analyzed using multiple algorithms. The results showed that activation of necroptosis contributed to myocardial dysfunction in the mouse model of SIMD generated by the lipopolysaccharide challenge. Our results confirmed that necroptosis was associated with changes in the immune microenvironment in SIMD.

Necroptosis plays a crucial role in tissue damage and inflammation, and participates in the pathophysiology of multiple human diseases (Kearney and Martin, 2017). Necroptosis is implicated in cardiac ischemia-reperfusion injury and myocardial depression induced by oxidative stress (Soppert et al., 2018; Heckmann et al., 2019). In the present study, GSEA showed that necroptosis was significantly upregulated in SIMD. The necroptosis pathway is triggered during pathogen infection and cellular damage by activation of specific death receptors including TNF receptor, FAS, and TLR3 (Vandenabeele et al., 2010). Our results demonstrated that the expression of necroptosis-related markers was increased in the heart tissues of SIMD mice. Furthermore, we confirmed the role of necroptosis in LPS-induced myocardial dysfunction. QRT-PCR and western blotting results showed that MLKL and RIPK3 were significantly upregulated in the hearts of SIMD model mice. Immunohistochemistry data was consistent with the RNA sequencing results. These data demonstrated that necroptosis-related genes are potential diagnostic biomarkers and therapeutic targets for patients with sepsis-induced myocardial dysfunction.

GSEA also identified other critical biological pathways related to immune response that are relevant to the understanding the mechanisms underlying SIMD. This included pathways related to apoptosis, NOD-like receptor (NLR) signaling pathway, Toll-like receptor signaling pathway and JAK-STAT signaling pathway. Apoptosis is a programmed cell death mechanism that is associated with sepsis-induced myocardial dysfunction (Cao et al., 2021). NLR mediates synthesis of intracellular inflammatory mediators and cytokines that participate in the inflammatory response. Recent studies have focused on the role of the NLRP3 inflammasome, a key component of the innate immune system, in SIMD. NLRP3 inflammasome triggers CLP-induced sepsis cardiomyopathy (Kalbitz et al., 2016). Furthermore, inhibition of the NLRP3 inflammasome protects against sepsis-induced cardiomyopathy (Busch et al., 2021). Toll-like receptors play a key role in the activation of necroptosis (Cao et al., 2018). For example, LPS activates necroptosis in murine macrophages through the TLR4 adapter protein, TRIF (He et al., 2011). TLR4 activation promotes release of pro-inflammatory cytokines and infiltration of neutrophils, thereby exacerbating cardiac damage caused by severe sepsis (Zhou et al., 2018). JAK-STAT pathway is the principal signal transduction pathway for various cytokines involved in sepsis (Cai et al., 2015). STAT1, a JAK-STAT signal transduction family member, is required for LPS-induced synthesis and secretion of pro-inflammatory factors. STAT1 knockout mice are resistant to LPS-induced endotoxemia and CLP-induced septic shock (Herzig et al., 2012). In the experimental sepsis model, hyperactivation of STAT1 and STAT3 in the macrophages and monocytes induces immune system dysfunction and excessive release of inflammatory factors, resulting in sepsis-induced heart injury. Immune dysfunction-related myocardial injury during infections can be suppressed or alleviated by modulating the JAK/STAT signaling pathways (Ashton et al., 2017).

In the present study, we observed upregulation of pyroptosis-related genes such as NLRP3 and IL-1β. During pyroptosis, activated caspase-1 or caspase-4/5/11 cleave and activate gasdermin (Gsdmd), a pore-forming protein that ruptures cell membranes and induces cell death (Chen et al., 2019). LPS activates pyroptosis *via* caspase 11, which is required for endotoxic shock in mice (Shi et al., 2014). RIPK3 is another essential effector molecule during necroptosis. Vesicular stomatitis virus (VSV) triggers serine-threonine kinases, RIPK1 and RIPK3, which promote mitochondrial damage by activating the DRP1-GTPase (Rayamajhi and Miao, 2014). This causes excessive generation of reactive oxygen species (ROS) and subsequent activation of the NLRP3 inflammasome. Moreover, Z-DNA-binding protein 1 (ZBP1), a novel effector of necroptosis, triggers NLRP3 inflammasome activation and release of IL-1β through the RIPK1-RIPK3-Caspase-8 axis during influenza A virus (IAV) infection (Huang et al., 2021). This showed that necroptosis effectors promote activation of the NLRP3 inflammasome. Furthermore, RIPK3 promotes NLRP3 inflammasome activation and IL-1β inflammatory responses independent of MLKL and necroptotic cell death (Lawlor et al., 2015). In our study, immunohistochemical analysis demonstrated activation of markers related to both necroptosis and pyroptosis. These results suggested that pathogenesis of SIMD involved activation of different types of programmed cell death pathways.

Our results showed that immune-related pathways such as the cytokine-cytokine receptor signaling pathway, B cell-mediated immunity, T cell-mediated immunity, and natural killer cell-mediated immunity were enriched in the SIMD group. CIBERSORT analysis demonstrated increased infiltration of monocytes, M1 macrophages, and neutrophils, and decreased infiltration of naive B cells and plasma cells in the heart tissues of the SIMD model mice. The progression of LPS-induced myocardial dysfunction involves gradual increase in the numbers of M1 macrophages and subsequent decrease in the numbers of M2 macrophages (Chagnon et al., 2005; Orecchioni et al., 2019). Our results were in concordance with these reports. Neutrophils are a subset of immune cells that play a key role in acute and chronic inflammation by trapping and killing invading pathogens (Kolaczkowska and Kubes, 2013). When infection is not contained locally during the initial immune response, it could result in dysregulated inflammatory response and cause organ damage and sepsis, which increases the risk of death (Vachharajani and McCall, 2019). During infection, neutrophils recruit monocytes to the site of inflammation (Liew and Kubes, 2019). Our study showed that B cells and plasma cells were significantly reduced in the heart tissues of SIMD mice. Reduction of B-cells is closely associated with unsatisfactory outcomes in sepsis (Monserrat et al., 2013). Systemic immune response plays a significant role in the pathogenesis of severe sepsis. Since inflammation is a significant cause of death or complications in sepsis, regulation of the immune system to cope with infection and maintain normal function has become a hotspot of infectious disease research (Hotchkiss et al., 2013). Our data showed significant differences in the expression levels of necroptosis-related genes and immune cell infiltration between the SIMD and control groups. This suggested correlation between necroptosis and the immune microenvironment in the pathogenesis of SIMD.

The relationship between necroptosis and immune infiltration is complex. Necroptosis is one of the cell death mechanisms in several types of immune cells. Alternatively, immune cells may recognize necroptotic myocardial cells and elicit a robust inflammatory response. Our study demonstrated significant correlation between necroptosis and immune cell infiltration in SIMD, but further studies are needed to validate our findings.

This study has several limitations. Firstly, this study involved sepsis-induced myocardial dysfunction model that was induced by intraperitoneal administration of LPS. The LPS model may not ideally represent human SIMD. Therefore, further pre-clinical and clinical studies are necessary to characterize the mechanisms underlying SIMD. Secondly, our study performed bioinformatics analysis of animal SIMD model samples. However, the sample size was small. Therefore, further extensive data analysis and samples are needed to verify our findings. Thirdly, the transcriptomic data used for immune infiltration analysis showed that necroptosis was related to immune cell infiltration. The immunohistochemistry experiments showed necroptosis in the myocardial cells. It is not clear from our results whether necroptosis of myocardial cells promoted immune cell infiltration or whether immune cell participation was not related to necroptosis. Therefore, further investigations are necessary to clarify the potential mechanisms through both *in vitro* and *in vivo* studies.

## Conclusions

In conclusion, this study demonstrated that LPS-induced inflammatory responses caused myocardial injury during sepsis. This study also demonstrated that necroptosis-related genes were associated with increased immune cell infiltration into the cardiac tissues in the SIMD model mice. We identified several necroptosis-related genes that may be potential diagnostic biomarkers and therapeutic targets for SIMD. These results provide a better understanding of the role of necroptosis, immune cell infiltration, and inflammation in the pathogenesis of SIMD.

## Data availability statement

The datasets presented in this study can be found in online repositories. The names of the repository/repositories and accession number(s) can be found in the article/supplementary material.

## Ethics statement

The animal study was reviewed and approved by the Ethics Committee of Southwest Medical University for Animal Care and Treatment (IACUC Issue NO.SWMU20220041).

## Author contributions

Study Design: YD, YZ, RD, and QP. Data Collection: YD, YZ, and QP. Statistical Analysis: YD, YZ, QZ, and QP. Data Interpretation: YD, YZ, and QP. Manuscript Preparation: YD, YZ, RD, QZ, and QP. Literature Search: YD, YZ, FX, XW, and QP. Funds Collection: QP and QZ. All authors contributed to the article and approved the submitted version.
